# A novel multilocus variable number tandem repeat analysis typing scheme for African phylotype III strains of the *Ralstonia solanacearum* species complex

**DOI:** 10.7717/peerj.1949

**Published:** 2016-05-05

**Authors:** Santatra Ravelomanantsoa, Isabelle Robène, Frédéric Chiroleu, Fabien Guérin, Stéphane Poussier, Olivier Pruvost, Philippe Prior

**Affiliations:** 1 BIOS UMR PVBMT, CIRAD, Saint-Pierre, La Réunion, France; 2 UMR PVBMT, Université de la Reunion, Saint-Denis, La Réunion, France; 3 Faculty of Sciences, University of Antananarivo, Antananarivo, Madagascar; 4 UMR PVBMT, CIRAD, Saint-Pierre La Réunion, France; 5 UMR PVBMT, Université de la Reunion, Saint-Pierre La Réunion, France; 6 Department of Plant Health and Environment, INRA, Paris, France

**Keywords:** *Ralstonia solanacearum* species complex, African phylotype III, MLVA, MLST

## Abstract

**Background.** Reliable genotyping that provides an accurate description of diversity in the context of pathogen emergence is required for the establishment of strategies to improve disease management. MultiLocus variable number tandem repeat analysis (MLVA) is a valuable genotyping method. It can be performed at small evolutionary scales where high discriminatory power is needed. Strains of the *Ralstonia solanacearum* species complex (RSSC) are highly genetically diverse. These destructive pathogens are the causative agent of bacterial wilt on an unusually broad range of host plants worldwide. In this study, we developed an MLVA scheme for genotyping the African RSSC phylotype III.

**Methods.** We selected different publicly available tandem repeat (TR) loci and additional TR loci from the genome of strain CMR15 as markers. Based on these loci, a new phylotype III-MLVA scheme is presented. MLVA and multiLocus sequence typing (MLST) were compared at the global, regional, and local scales. Different populations of epidemiologically related and unrelated RSSC phylotype III strains were used.

**Results and Discussion.** Sixteen polymorphic TR loci, which included seven microsatellites and nine minisatellites, were selected. These TR loci were distributed throughout the genome (chromosome and megaplasmid) and located in both coding and intergenic regions. The newly developed RS3-MLVA16 scheme was more discriminative than MLST. RS3-MLVA16 showed good ability in differentiating strains at global, regional, and local scales, and it especially highlighted epidemiological links between closely related strains at the local scale. RS3-MLVA16 also underlines genetic variability within the same MLST-type and clonal complex, and gives a first overview of population structure. Overall, RS3-MLVA16 is a promising genotyping method for outbreak investigation at a fine scale, and it could be used for outbreak investigation as a first-line, low-cost assay for the routine screening of RSSC phylotype III.

## Introduction

Genotyping with appropriate genetic markers (i.e., markers adapted for the evolutionary scale being investigated) is a valuable tool that has been effectively used to study infectious diseases, resolve the epidemiology of pathogens, and improve disease management strategies (Maiden et al., 1998; Michael, 2001; Coloma & Harris, 2009; Harrington et al., 2014). Disease outbreaks pose a serious and continuing threat to sustainable agriculture throughout the world. Genotyping is a rapidly developing molecular approach that can be used to address the increasing threats from crop disease (Fox & Narra, 2006; Vinatzer & Bull, 2009). Portable and highly resolved deoxyribonucleic acid (DNA) sequence-based typing methods have been extensively investigated and successfully applied to characterize the causative agents of plant diseases, their transmission pathways, and their spatiotemporal expansion. These studies ideally use whole-genome sequence data to provide precise and robust genomic information regarding genetic variation within individuals. However, a simple and cost-effective genotyping method is needed for routine use in disease outbreak investigations.

Multilocus sequence typing (MLST) and multilocus sequence analysis (MLSA) typically determine sequence polymorphisms in 7–8 housekeeping genes. These genotyping methods are consistently used to infer genetic relationships and evolutionary history on a global scale. Therefore, these methods are recognized as the gold standard for the global epidemiology of bacterial pathogens (Maiden, 2006). However, genotyping techniques with higher discriminatory power are useful for investigations at small evolutionary scales and the study of monomorphic pathogens (Achtman, 2008).

Multilocus variable number tandem repeat analysis (MLVA) is an alternative genotyping method that targets tandem repeats (TRs), which are some of the most polymorphic loci found in genomes (Van Belkum et al., 1998). Variation in TRs may be generated because of DNA polymerase slippage during replication and/or unequal recombination at TR loci (Jeffreys, 1987). TRs are subdivided according to the sizes of the repeat units: microsatellites contain 1–6 bp repeat units and minisatellites contain 7–60 bp repeat units (Vergnaud & Pourcel, 2009). TRs can be perfect (an uninterrupted array of motif copies) (Weber, 1990; Kashi & King, 2006), imperfect (an array with some variation in the sequence of one or more of the repeating motifs) (Kashi & King, 2006), or compound (an array containing various repeating motifs) (Weber, 1990). MLVA is used to assess the variation in the TR copy number at multiple loci to differentiate isolates. MLVA is increasingly used to resolve the genetic relatedness among closely related strains at micro-evolutionary levels (Lindstedt, 2005; Lindstedt et al., 2013).

Bacterial wilt caused by the *Ralstonia solanacearum* species complex (RSSC) is one of the most destructive plant diseases worldwide. RSSC affects a wide range of host species—primarily vegetable, woody, and ornamental species. This soil-borne and xylem-limited bacterium is an unusually heterogeneous and highly disparate organism (Hayward, 1964; Gillings & Fahy, 1993; Prior, Allen & Elphinstone, 1998; Fegan & Prior, 2005); most likely it is naturally competent for transformation ( Coupat et al., 2008) and recombination (Wicker et al., 2012). The RSSC is genetically differentiated into four major phylogenetic lineages, which are known as phylotypes. The phylotype designation is related to the geographical origin of strains: phylotype I contains strains from Asia, phylotype II contains strains from the Americas, phylotype III contains strains from Africa and the Indian Ocean, and phylotype IV contains strains from Indonesia, Japan, and Australia (Prior & Fegan, 2005). The characterization of RSSC isolates at both broad and small spatial scales is crucial for understanding the biology and epidemiology of bacterial wilt disease. This characterization is necessary for the development of effective control strategies.

Few population and evolutionary genetics studies have used RSSC populations to investigate bacterial wilt outbreaks in fine-scale geographical areas. For this purpose, MLSA (Wicker et al., 2012) and phylotype-specific MLVA schemes have been developed (N’Guessan et al., 2013; Parkinson et al., 2013).

Poussier, Vandewalle & Luisetti (1999) first described the RSSC strains in phylotype III. To date, these strains have only been isolated in a few countries in Sub-Saharan Africa (Angola, Burkina Faso, Cameroon, Guinea, Ivory Coast, Kenya, and Zimbabwe) and the Southwest Indian Ocean (Madagascar and Reunion). Phylotype III strains cause bacterial wilt epidemics, which impact food security by compromising crops and livelihoods at the household and national levels. However, the population biology of phylotype III strains remains poorly investigated and poorly understood. Knowledge of their population biology is necessary to resolve important aspects of bacterial wilt control, especially the management of resistant cultivars. Among all of the publicly available RSSC genomes (∼40), the genome of only one phylotype III strain (CMR15) (Remenant et al., 2010) is currently available. Only a few phylotype III strains are maintained in international collections.

In this work, we first evaluated the RS3-MLVA11 scheme developed by N’Guessan et al. (2013), which was designed to type RSSC phylotype III. This scheme was based on 11 TR loci selected from strains belonging to different RSSC phylotypes (I, IIA, IIB, III, and IV) and was tested on a limited collection of isolates (N’Guessan et al., 2013). We demonstrated that the RS3-MLVA11 scheme was not completely appropriate for the study of the genetic structure of RSSC phylotype III populations. We developed an improved MLVA scheme specific for RSSC phylotype III. Compared to MLST, this MLVA achieved better resolution by subtyping MLST clones when performing investigations at a small spatiotemporal scale.

## Materials and Methods

### Bacterial strains and populations

Three populations of RSSC phylotype III isolates were used (Table S1). All 65 isolates used in this study were pooled in collection C65. This is a diverse collection that includes epidemiologically unrelated strains and closely related strains from the same country and the same outbreak. Population P35 is a worldwide population composed of 35 unrelated isolates originating from Africa (Angola, Burkina Faso, Cameroon, Guinea, Ivory Coast, Kenya, and Zimbabwe) and the Southwest Indian Ocean (Reunion and Madagascar); these strains were isolated from various host species. This population includes isolates used in previous MLVA (*n* = 21) and MLSA (*n* = 14) studies (N’Guessan et al., 2013; Wicker et al., 2012). Population P20 contains 20 isolates collected in 2005 from 3 different agro-ecological zones (AEZ3, AEZ4, and AEZ5) in Cameroon; these strains were isolated from garden huckleberry (*Solanum scabrum*), tomato (*Solanum lycopersicum*), potato (*Solanum tuberosum*), and pepper (*Capsicum annuum*) (Mahbou Somo Toukam et al., 2009). Population P17 contains 17 epidemiologically related isolates recovered in 2005 from tomatoes grown in a single field in Cameroon (Mfou, AEZ3) (Table S1).

Isolates were routinely grown at 28 °C on Nutrient Broth, Kelman’s triphenyl tetrazolium chloride agar medium (Kelman, 1954), and modified semi-selective agar medium from South Africa (Elphinstone et al., 1996). A 1-µl loop was used to collect fresh overnight colonies from agar plates. Cells were suspended in 200 µl of sterile HPLC-grade water and used as templates for PCR amplification.

### Design of the optimized MLVA scheme

The design of the optimized MLVA scheme involved the following three major steps: the TR loci to be characterized were selected, the primers for gene amplification were designed, and the multiplex PCR reactions were optimized.

#### Examination of previous TR loci (RS3-MLVA11 scheme)

The RS3-MLVA11 scheme (N’Guessan et al., 2013) consisted of the following 11 TR loci: RS2AL03, RS1L05, RS1L09, RS1L10, RS1L12, RS3L17, RS3L18, RS3L19, RS3L20, RS2BL23, and RS4L26. These markers were designed using publicly available genomes of strains belonging to different RSSC phylotypes.

RS2AL03 was identified from the phylotype IIA strain CFBP2957. RS2BL23 was identified from the IIB strain MOLK2. RS1L05, RS1L09, RS1L10, and RS1L12 were identified from the phylotype I strain GMI1000. RS4L26 was identified from the phylotype IV strain PSI07. RS3L17, RS3L18, RS3L19, and RS3L20 were identified from the phylotype III strain CMR15. The average nucleotide identity (ANI) values are as follows: 91.2% between CMR15 and CFBP2957 (IIA-36), where RS2AL03 is identified; 90.9% between CMR15 and Molk2 (IIB-3), where RS2BL23 is identified; 96.3% between CMR15 and GMI1000 (I-18), where RS1L05, RS1L09, RS1L10, and RS1L12 are identified; and 92.4% between CMR15 and PSI07 (IV-10), where RS4L26 is identified (Remenant et al., 2010). We examined the presence and structure of TR loci identified from strains outside phylotype III (RS2AL03, RS2BL23, RS1L05, RS1L09, RS1L10, RS1L12, and RS4L26) in the genome of the reference phylotype III strain CMR15. In silico analyses were performed using the Geneious v7.1.7 software package (Biomatters, Auckland, New Zealand). Sequence alignments were performed for each locus to verify the presence of the TRs in the chromosome or megaplasmid replicons of CMR15. Sequence alignments were also used to confirm that the configuration of each locus and its repeat units met the following protocol requirements: the lengths of the PCR products were between 100 and 500 bp for matching the ladder used during multiplex capillary electrophoresis, the TR sequence was not duplicated in the genome, typeability reached 100% for phylotype III strains, and the sequence identity within the TR array was greater than 80%, as recommended (Vergnaud & Pourcel, 2009). This recommendation is because variation at TR loci with short repeat units is highly dependent upon the homogeneity of the repeat stretches (Pourcel & Vergnaud, 2011).

#### Identification of new TR loci

We used the complete genome of the phylotype III strain CMR15 to identify new TR loci (accession numbers FP885895 for the chromosome and FP885896 for the megaplasmid). The CMR15 genome was screened for repetitive DNA sequences using the Tandem Repeats Finder software package (https://tandem.bu.edu/trf/trf.html) (Benson, 1999). Candidate TRs were selected according the following criteria. *(i)* No *indel* occurred between adjacent copies. *(ii)* The sequence identity within the TR array was greater than 85%. *(iii)* The size of the consensus pattern was ≤20 bp. *(iv)* The copy number was greater than 3. In the event of multiple TR assignations reported for a same locus, the shorter repeat was retained. *(v)* Regions containing overlapping or successive TR arrays were not considered. *(vi)* The TR was found only once within the genome. *(vii)* The PCR products ranged in length from 100 to 500 bp for matching the ladder used during multiplex capillary electrophoresis.

#### Primer design and assay optimization

Primer pairs flanking the TR candidates were designed using the Primer3 v4.0.0 software package (http://primer3.ut.ee/) (Untergasser et al., 2012). The conditions for selecting the primers were the primer size (18–23 bp), melting temperature (Tm; 57–65 °C), and guanine-cytosine (GC) content (40–70%). Primers with a low probability of dimer or hairpin loop formation and product sizes ranging between 100 and 500 bp were selected. The potential for secondary structure formation and dimerization was assessed using the OligoAnalyzer v3.1 software package (https://eu.idtdna.com/calc/analyzer) (OligoAnalyzer^®^ Tool; IDT, Coralville, IA, USA). Oligonucleotides were synthesized by Macrogen, Inc. (Seoul, Korea). The primers were tested via simplex PCR using RSSC isolates from population P35. PCR amplification was performed in 15-µl reaction volumes containing 7.5 µl 2 × QIAGEN Type-it Multiplex PCR Master Mix, 3 µl 5 × Q-solution (Qiagen, Hilden, Germany), 1.5 µl of a forward and reverse primer mix (2 µM each), 2 µl sterile HPLC-grade water, and 1 µl of a bacterial suspension as a template. The PCRs were performed in a GeneAmp^®^ PCR System 9,700 thermal cycler (Applied Biosystems, Foster City, CA, USA) using the following conditions: an initial denaturation step at 95 °C for 5 min; 30 cycles of denaturation at 95 °C for 30 s, annealing at a temperature gradient (57, 60, 62, and 64 °C) for 90 s, and extension at 72 °C for 30 s; and a final extension step at 60 °C for 30 min. Six microliters of the PCR product were mixed with 1 µl of loading dye solution and loaded into a 1.5% (w/v) SeaKem^®^ LE Agarose (Lonza, Basel, Switzerland) gel for electrophoresis. The gels were stained with ethidium bromide. The bands were visualized/photographed under ultraviolet (UV) light using the G:BOX gel imaging system (Syngene, Cambridge, UK). The molecular weights were estimated by comparison with a 100-bp DNA ladder (Promega, Madison, Wisconsin, USA). Loci with poor amplification and/or that lacked diversity were discarded. Sequences of the primers used to amplify previous TR loci were obtained from a previous report (N’Guessan et al., 2013); one exception was RS1L12, which was redesigned to improve amplification. The primer nomenclature has been adapted to the nomenclature for oligonucleotide probes described previously (Alm et al., 1996). Descriptions of the TR loci and the corresponding 5′-labelled and unlabelled primer sets used in this study are presented in Table S2.

According to the amplicon size, four sets of primer mixes, which consisted of four specific primer pairs each, were created for the simultaneous amplification of multiple TR loci (multiplex PCR) (Table S2). The suitability of the primer combinations was evaluated via PCR, and the band profiles generated were assessed via gel electrophoresis. The multiplex PCR protocol was performed as described for simplex PCR, with some modifications. The reactions included 1.5 µl of a primer mix containing four primer pairs (2 µM each), and the optimal annealing temperature for each set of primer mixes was 62 °C.

### MLVA genotyping

Prior to the size analyses, 1 µl of fluorescent PCR products was diluted in sterile HPLC-grade water; the dilutions were between 1:10 and 1:50, as determined from the test runs. The diluted aliquots (1 µl) were mixed with 10.7 µl Hi-Di formamide (Applied Biosystems) and 0.3 µl GeneScanTM-500 LIZ^®^ Size Standard (Applied Biosystems). The samples were denatured at 95 °C for 5 min and cooled immediately on ice for 5 min before being loaded onto a 3130xl Genetic Analyzer. Capillary electrophoresis was performed using 36-cm capillaries filled with POP7-polymer (Applied Biosystems). The assays were run at 60 °C for 30 min, with a running voltage of 15 kV. Injection was performed at 15 kV for 23 s. Each TR locus was identified as a peak according to its colour and size (Table S2). Peaks were assigned a size with the GeneMapper^®^ v4.0 software package (Applied Biosystems) using the settings for microsatellite analysis.

The amplicon sizes were converted into repeat numbers. The repeat numbers for two to four alleles (depending on the range of repeat numbers) from each locus were confirmed by sequencing. The sequence analysis results were also used to check for patterns in the flanking sequences and internal repeat variations (i.e., copy homology). The calculated number of repeats, referred to as alleles, were combined into a string and ordered according to the position of the loci in the CMR15 genome. The allele strings were reported as MLVA profiles. Each unique MLVA profile was given a haplotype designation, called an MLVA type (MT), using the GenAlEx v6.5 software package (Peakall & Smouse, 2012). Each isolate tested was assigned an MT. Isolates that differed by one or more alleles were considered distinct types. The MLVA profiles were used for comparison and clustering.

### MLST genotyping

Seven gene loci (*gdhA*, *mutS*, *adk*, *leuS*, *rplB*, *gyrB*, and *egl*) were chosen from a previous MLSA scheme (Wicker et al., 2012). These genes were the most discriminative markers, and less recombination was observed within the phylotype III lineage. MLST was performed for isolates in collection C65. These genes were partially amplified using sets of primers described previously (Wicker et al., 2012); the *gyrB* primers were redesigned to address amplification failures with some isolates. Descriptions of the loci and the corresponding oligonucleotide primer sets are presented in Table S3. Amplification was performed in 50-µl reaction volumes consisting of 10 µl 5 × Colorless GoTaq Flexi Buffer (Promega), 3 µl MgCl_2_ solution (25 mM), 1 µl dNTP mix (10 mM each), 1.25 µl of a primer pair mix (10 µM each), 0.25 µl of GoTaq^®^ G2 Flexi DNA Polymerase (5 µ.µl^−1^), 31.25 µl sterile HPLC-grade water, and 2 µl of fresh bacterial suspensions used as a template. The optimized amplification protocol was as follows: an initial denaturation at 96 °C for 9 min; 30 cycles of denaturation at 95 °C for 1 min, an appropriate annealing temperature (Table S3) for 90 s, and elongation at 72 °C for 90 s; and a final extension at 72 °C for 10 min. The amplified PCR products were separated on 1.5% agarose gel to visualize the amplification quality. Samples of sufficient quality were sent to Beckman Coulter Genomics for DNA double-strand sequencing (forward and reverse). The primers used for PCR were also used for sequencing. The raw sequence data were edited using the Geneious v7.1.7 software package. The consensus sequences for each sequenced gene were determined by assembling forward and reverse chromatograms. The sequences corresponding to primer pairs and ambiguous 5′ and 3′ sequences were discarded from the analysis; this was performed to avoid sequence bias and to ensure correct sequence analysis. The consensus sequences were then aligned using Muscle in Geneious v7.1.7. At each locus, each unique sequence was considered an allele. Each strain was assigned an MLST profile containing seven allele numbers. Each unique MLST profile was given an MLST type (ST) designation. The MLST profiles were used for comparison and clustering.

A total of 98 MLST loci sequences used in this study were retrieved from GenBank; 357 newly generated sequences were deposited in GenBank under the accession numbers KU255860 through KU256216. The accessions numbers of sequences are listed in Table S4.

### Data analysis

All analyses were performed with R v 3.0.2 software package (R Core Team, 2013), except where other software is indicated. The typeability (T) of each marker locus was defined as the amplification success rate. The level of polymorphism was evaluated for each locus per population (P17, P20, and P35) and for the collection C65 combination using the GenAlEx v6.5 software package by computing the number of alleles per locus (Na), the percentage of polymorphic markers, the allelic range (AR), and *Nei’*s marker of diversity index (*H*_*E*_). The allelic richness (A) estimates the genetic diversity in a population; it was calculated per locus and population using the *allelic.richness* function in the “hierfstat” package (Goudet, 2014). This calculation uses rarefaction to measure the number of alleles per locus in a random subsample of uniform size (*n* = 17) drawn from the population.

The resolution of the set of TR loci was evaluated by computing the number of unique haplotypes observed for all possible combinations of *k* loci (*k* = 1–16). A haplotype accumulation curve (HAC) for increasing numbers of loci was generated for each population tested. Reaching a plateau indicated that the locus set tested was sufficient to identify all unique haplotypes (Arnaud-Haond et al., 2005).

MLVA typing performance was compared to MLST. The discriminatory power of typing systems was evaluated by calculating the Hunter Gaston Discrimination Index (HGDI) (Hunter & Gaston, 1988) using the Discriminatory Power Calculator tool available at http://insilico.ehu.es/mini_tools/discriminatory_power/ (Hunter & Gaston, 1988; Hunter, 1990). This index measures the probability that two randomly sampled strains from a population will have different haplotypes. Congruence between MLVA and MLST methods was calculated using the adjusted Rand coefficient (aR) and the adjusted Wallace coefficient (aW) and jackknife pseudo-values 95% confidence interval (CI) with the Comparing Partitions tool at http://darwin.phyloviz.net/ComparingPartitionsv2/index.php?link=Tool. aR indicates the agreement between the two typing methods. aW indicates the agreement between partitions and gives the probability that for a given data set, a pair of strains grouped in the same type under a method is also grouped as identical under another method (Severiano et al., 2011). The strength of correlation between distance matrices was confirmed using the Mantel test (Mantel, 1967); similarities were determined using the *cadm.post* function (Legendre & Lapointe, 2004) provided by the “ape” package. Statistical significance was assessed using 10,000 permutations of a single matrix.

Finally, the ability of MLST and MLVA genotyping to link or differentiate strains was assessed. MLVA and MLST minimum spanning trees (MSTs) for collection C65 were analysed. MSTs were built using the goeBURST full MST algorithm, which is based on the Euclidean and goeBURST distances between two profiles; this was implemented in the PHYLOViZ v1.0 software package (Francisco et al., 2012). Clonal complexes (CCs) were defined as groups of genetically related haplotypes and linked by a single locus variant (i.e, that vary at a single locus - SLV).

## Results and Discussion

### Evaluation of the TR markers from the RS3-MLVA11 scheme

Among the 11 TR loci previously described for phylotype III strains (N’Guessan et al., 2013), seven TR loci originally described from non-phylotype III strains were examined for their presence and structure in the genome of the reference phylotype III strain CMR15. Tandem Repeats Finder did not identify any TRs in the CMR15 regions matching RS2AL03 (Fig. S1A) and RS2BL23 (Fig. S1B). The repeat and flanking regions from RS1L09 and RS3L18 were highly homologous, suggesting that N’Guessan et al. (2013) used two loci designed from a phylotype I and phylotype III strain, respectively, but corresponding to the same single locus. In addition, they consisted of two consecutive and distinct TR arrays with respective tandem repeat units of 9 and18 bp, respectively (Fig. S1C). The inclusion in an MLVA scheme of tandem-repeat arrays composed of two or more completely different tandem repeat motifs should be avoided for a purpose where the evolution mode of the markers should be deciphered. Moreover, such a dual source of polymorphism would strongly increase the risk of homoplasy. For example, a difference of two 9-bp repeats is evolutionarily dissimilar from a single-repeat variation of an 18-bp motif, which will likely have a different evolution rate. RS4L26 was not selected, as we confirmed in previous data from N’Guessan et al. (2013) suggesting that no amplicon could be produced from a majority of strains in C65. Despite multiple attempts, we failed to design optimized primers for RS4L26 to meet our selection requirements. The RS3L20 locus was partitioned into complex sub-sequences of TR motifs with only 51% mean identity (Fig. S2). Finally, new primer sets were developed for RS1L12 to address the amplification failure reported in the original study (N’Guessan et al., 2013) (Table S2). We observed that homologous TR loci originating from different phylotypes did not always have the same internal structure.

In this study, we retained the following five TR loci from RS3-MLVA11: RS3L17, RS3L19, RS1L05, RS1L10, and RS1L12. These five TR loci fulfilled our selection criteria and were successfully amplified from collection C65 (100% typeability) (Table 1).

**Table 1:** Measures of genetic variability using TR and MLST loci. Typeability (T), allelic range (AR), observed allele number (Na), allelic richness (A), Nei’s unbiased diversity index (*HE*). Typeability = 100%. All strains were typed at all loci. Hunter-Gaston Diversity Index (HGDI): power of discrimination for the molecular typing methods.

VNTR loci	AR	Collection												MLST loci	AR	Collection											
		P35			P20			P17			P65					P35			P20			P17			P65		
		Na	A	*H*_*E*_	Na	A	*H*_*E*_	Na	A	*H*_*E*_	Na	A	*H*_*E*_			Na	A	*H*_*E*_	Na	A	*H*_*E*_	Na	A	*H*_*E*_	Na	A	*H*_*E*_
RS3L27^a^	2–11, 13, 37	10	8.23	0.89	5	4.97	0.77	1	1	0.00	12	7.03	0.84	gdhA	1–17	17	10.87	0.93	4	3.58	0.36	1	1	0.00	17	7.93	0.84
RS3L28^a^	2–5	4	3.30	0.39	2	2.00	0.34	2	2	0.12	4	2.97	0.54	gyrB	1–16	14	9.02	0.89	7	6.25	0.73	1	1	0.00	16	7.89	0.85
RS3L29^b^	2–3	2	2.00	0.44	2	1.98	0.19	1	1	0.00	2	2.00	0.51	rplB	1–18	18	11.59	0.94	4	3.68	0.36	1	1	0.00	18	8.15	0.84
RS3L17^a^	3, 7–15, 18–20	12	9.26	0.91	6	6.67	0.84	1	1	0.00	13	8.17	0.81	leuS	1–16	15	10.18	0.92	4	3.68	0.36	1	1	0.00	16	7.76	0.83
RS3L19^b^	3–10, 15	8	6.38	0.78	5	4.55	0.60	1	1	0.00	9	5.82	0.64	adk	1–15	14	10.15	0.93	5	4.69	0.69	1	1	0.00	15	8.30	0.85
RS3L30^b^	3–4, 8–9, 12–13, 19, 25–27, 30, 34, 40	12	8.21	0.86	3	3.00	0.64	3	3	0.32	13	7.08	0.79	mutS	1–22	20	12.22	0.95	6	5.53	0.74	1	1	0.00	22	9.48	0.88
RS3L31^a^	2, 6	2	1.74	0.11	2	1.98	0.19	1	1	0.00	2	2.00	0.42	egl	1–20	19	11.55	0.94	5	4.53	0.44	1	1	0.00	20	8.32	0.85
RS3L32^a^	2–3	2	2.00	0.39	2	2.00	0.34	1	1	0.00	2	2.00	0.50														
RS3L33^a^	6, 8–9, 12–13, 15–16, 18–20, 22, 26–27, 35	14	9.33	0.89	7	6.38	0.73	1	1	0.00	14	6.65	0.69														
RS3L34^b^	4, 6–10	6	5.05	0.62	3	3.00	0.67	1	1	0.00	6	4.37	0.68														
RS1L05^b^	2–3	2	1.94	0.21	1	1.00	0,00	1	1	0.00	2	1.71	0.12														
RS3L35^b^	2, 4–7, 9	4	2.85	0.26	3	2.70	0.19	1	1	0.00	6	2.65	0.02														
RS3L36^b^	2–8, 10–11	9	7.86	0.90	4	3.85	0.65	1	1	0.00	9	6.36	0.78														
RS1L10^b^	3–5, 8–9	5	4.41	0.72	1	1.00	0.00	1	1	0.00	5	3.75	0.48														
RS3L37^a^	4–8, 10, 12–14, 16–17, 32, 36, 38	12	8.89	0.90	8	7.38	0.85	1	1	0.00	14	8.09	0.87														
RS1L12^a^	2, 9–18, 22	9	7.64	0.88	8	7.37	0.81	1	1	0.00	11	7.46	0.81														
Number of isolates		35			20			17			65			Number of isolates		35			20			17			65		
Polymorphic loci (%)		100			87.50			12.50			100			Polymorphic loci (%)		100			100			0			100		
Mean A		5.57			3.73			1.19			4.88			Mean A		10.80			4.58			1.00			8.26		
Mean *H*_*E*_		0.63			0.47			0.03			0.61			Mean *H*_*E*_		0.93			0.53			0.00			0.85		
HGDI		0.99			1.00			0.42			0.95			HGDI		0.98			0.91			0.00			0.91		
Haplotypes		32			20			4			48			Haplotypes		26			11			1			32		
Singletons		29			18			0			39			Singletons		22			3			0			22		

**Notes.**

a Loci located within intergenic regions.

b Loci located within coding regions.

### Identification of new TR markers

Additional TR loci were required to improve the resolution of MLVA typing. Because size homoplasy (i.e., alleles with distinct origins and the same state or length) is inherent to the TR loci, the use of a large number of TR loci could decrease the effect on genetic structure and population genetics indices (Estoup, Jarne & Cornuet, 2002; Reyes, Chan & Tanaka, 2012). Eighteen new TR loci were selected by screening the genome sequence of the phylotype III strain CMR15. Among these, 11 loci were successfully amplified from all isolates in collection C65 and exhibited size polymorphism (Table S2).

### Sixteen candidate markers for the new MLVA scheme

In total, 16 loci (five from RS3-MLVA11 and 11 newly identified) were selected as candidate markers for the new MLVA scheme (Table S2). A total of 41 DNA sequences for the alleles from the 16 TR loci were examined. It was confirmed that the differences in length of the different alleles were associated with differences in repeat unit copy numbers and not with indels in the flanking regions, either within or between the TR patterns. Local sequence alignment of the upstream and downstream flanking sequences of the 16 loci contained a few single-nucleotide polymorphisms. Furthermore, the majority of TR patterns among loci (80%) consisted of imperfect repeats. An example of a locus sequence analysis is provided in Fig. S3 for illustration. Nucleotide polymorphisms within the TR array could be used to complement a length polymorphism analysis (i.e., molecularly accessible size homoplasy) (Estoup, Jarne & Cornuet, 2002) to confirm the molecular evolution of the strains under study (Amonsin et al., 2004; Ablordey et al., 2005; Pradhan et al., 2011).

The newly developed MLVA scheme for phylotype III strains is hereafter referred to as RS3-MLVA16. This scheme consists of 16 TR loci regularly scattered throughout the chromosome (RS3L27-RS3L28-RS3L29-RS3L17-RS3L19-RS3L30-RS3L31-RS3L32) and on the megaplasmid (RS3L33-RS3L34-RS1L05-RS3L35-RS3L36-RS1L10-RS3L37-RS1L12) of the phylotype III strain CMR15 (Table S2). Seven loci were microsatellites (ranging from 5 to 6 bp), and nine loci were minisatellites (ranging from 7 to 18 bp). All of the retained TRs were mapped either inside coding sequences or in intergenic regions. Full descriptions of the TR loci and the corresponding oligonucleotide primers used in this study are provided in Table S2.

The same MLVA scheme can be used for addressing different questions. The required precision level of a genotyping technique is typically objective dependent. The analysis of a few isolates during an outbreak investigation would require a much lower refinement in the markers’ characteristics than a more complex population genetics analysis for which the evolution mode of the markers would need to be accurately determined. The original design of an MLVA scheme should allow the accomplishment of various objectives and consequently a thorough selection of markers should be implemented. Ideally, a single typing technique answering every investigator’s needs is desirable because it could bring together communities that have different, non-overlapping objectives. In this study, we provide an MLVA scheme optimized for practicality and containing a sufficiently high number of markers for maintaining homoplasy at a reasonable level, while eliminating markers for which future users could not appropriately evaluate their evolution mode and mutation rate.

### Discriminatory power of the newly developed RS3-MLVA16 scheme

**Figure 1:**
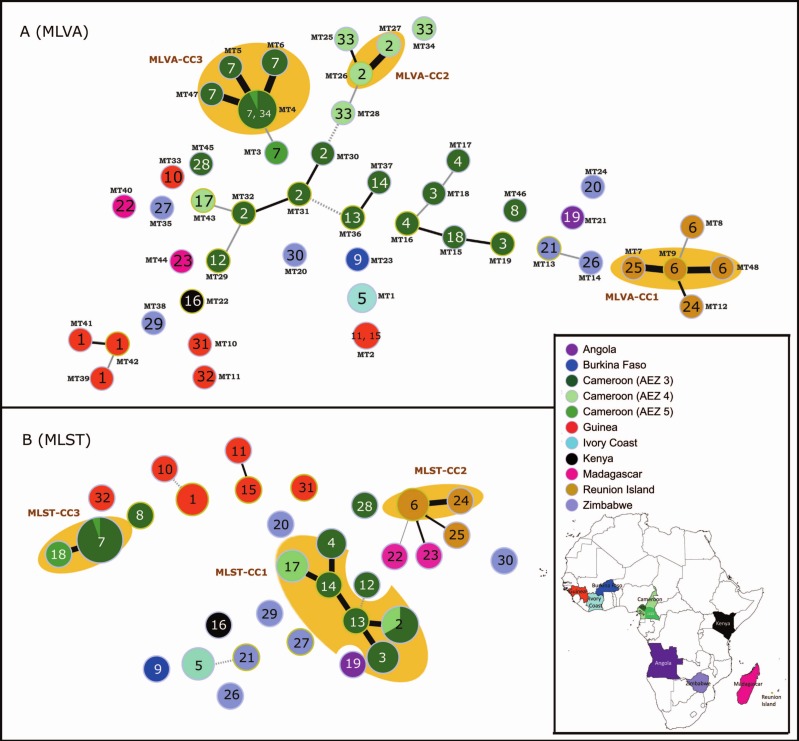
MSTs of *Ralstonia solanacearum* species complex strains of phylotype III (65 strains). (A) Data from the RS3-MLVA16 data. The dot diameter represents the number of strains per haplotype. (B) Data from the MLST analysis based on seven gene regions (*gdhA*, *mutS*, *adk*, *leuS*, *rplB*, *gyrB*, and *egl*). The dot colours indicate the country of isolation. The labels in the dots indicate the MLST sequence type numbers. The labels outside the dots indicate the MLVA type (MT) numbers. Black thick lines, black regular lines, grey thin lines, and grey dashed lines joining haplotypes indicate single-, double-, triple-, and quadruple-locus variations, respectively. No link is indicative of variations at >4 loci. Orange halos denote a clonal complex (CC).

At a global scale (P35; Africa and islands of the Southwest Indian Ocean), all loci were polymorphic with high indexes of diversity (*H*_*E*_ ranging from 0.11 to 0.91; mean *H*_*E*_: 0.63 and mean A: 5.57). RS3-MLVA16 showed high discrimination between isolates from different countries (HGDI: 0.99). The set of 16 loci discriminated 32 haplotypes with 29 singletons by Minimum Spanning Tree (Table 1), indicating that P35 was a diverse population and that the majority of haplotypes are epidemiologically unrelated between countries; however some haplotypes from Reunion were SLV in MLVA-CC1 (MT7, MT9, MT48) (Fig. 1A). The singletons were haplotypes for which no SLV could be identified in the population. Seven loci (RS3L17, RS3L37, RS3L36, RS3L27, RS3L33, RS3L12, and RS3L30) were identified as the most polymorphic; these loci had *H*_*E*_ values greater than 0.80 (Table 1). The large proportions of singletons and multiple alleles per locus highlighted the broad diversity among unrelated RSSC phylotype III isolates originating from different countries in Africa and the Southwest Indian Ocean (P35).

At a regional scale (P20; 3 AEZs in Cameroon), geographically related isolates were discriminated well using the 16 loci (*H*_*E*_ ranging from 0.00 to 0.85; mean *H*_*E*_: 0.47 and mean A: 3.73). Every isolate had a unique haplotype; of these, 18 were singletons (HGDI: 1) (Table 1). Within the country, six loci (RS3L37, RS3L30, RS3L12, RS3L27, RS3L17, and RS3L36) exhibited high polymorphism (*H*_*E*_ values ranging from 0.67 to 0.85), as was found at the global scale. Two loci (RS1L10 and RS1L05) were monomorphic (Table 1). These loci might be common between RSSC phylotype III strains from Cameroon; variations were observed among the broader population of African RSSC phylotype III strains (P35) and in the study performed by N’Guessan et al. (2013). Two haplotypes (MT26 and MT27) were epidemiologically related (MLVA-CC2) in AEZ4, whereas the other haplotypes were greater than SLVs between and within AEZs (Fig. 1A).

At a local scale (P17, one field in Cameroon), most RS3-MLVA16 loci were monomorphic (*n* = 14) among isolates (Table 1). The 16 loci suggested that genetic diversity was very narrow (*H*_*E*_ ranging from 0.00 to 0.32; mean *H*_*E*_ = 0.03 and mean A: 1.19). However, these loci clearly resolved 4 haplotypes (MT4, MT5, MT6, and MT47) without any singleton (HGDI: 0.42), suggesting the existence of outbreak-evolved strains that formed a complex clonal (MLVA-CC3) within P17 (Table 1 and Fig. 1A). MT5, MT6, and MT47 were variants of MT4.

The level of polymorphism of each locus and the discriminatory ability of RS3-MLVA16 differed at the three scales considered. The numbers of alleles, in descending order, were higher in populations P35, P20, and P17. This finding is consistent with the geographic origin of the strain members of each population. No differences in the levels of polymorphism were observed between loci from coding regions and those from intergenic regions; the variability could be associated with the evolutionary rate at each locus.

Altogether, the 65 isolates (C65) were resolved in 48 haplotypes by RS3-MLVA16, and these haplotypes contained 39 singletons and 3 clonal complexes (MLVA-CC1, MLVA-CC2 and MLVA-CC3) that each consisted of epidemiologically related strains (Fig. 1A). The discriminatory index (HGDI) of RS3-MLVA16 was 0.95. Strains from AEZ5 and the single field (Mfou) from Cameroon shared the same haplotype MT4, suggesting the introduction of MT4 from AEZ5 into the Mfou field. Haplotypes differed by 5 to 10 loci between countries and were SLV to 4 locus variants (LVs) within a country.

The resulting HACs of the locus combinations reached a plateau in all of the populations tested (Fig. S4). This result suggested that the 16 TR loci provided a good estimate of unique haplotypes and were sufficiently powerful to discriminate among RSSC phylotype III strains at both regional and local scales. A simplified MLVA typing scheme could be identified for routine epidemiological investigations and surveillance when RSSC phylotype III diversity is well known. The HAC indicates the minimum number of loci necessary to accurately resolve genetic differentiation in a given population. Based on the HACs, 4 loci generated 32 haplotypes for global population P35, 4 loci resolved 20 haplotypes for regional population P20, and 2 loci resolved 4 haplotypes for population P17. When all of the strains used in this study were considered (C65), 48 haplotypes were resolved using only 7 TRs (Table S5 and Fig. S4). We observed that the number of loci and the composition of the locus set depend on the population studied. This approach may be used by under-resourced laboratories. However, reducing the number of TR loci used for genotyping results in limitations due to the constraints of size homoplasy. As such, extreme caution should be taken when reducing the locus number; reductions can result in the loss of information concerning the true genetic relatedness of haplotypes. To overcome this problem, we recommend the use of 16 loci for genotyping strains from RSSC phylotype III.

### MLST data

All MLST gene fragments were successfully amplified for the 65 isolates used in this study.

At a global scale (P35), high polymorphism was revealed for each of the 7 loci (*H*_*E*_ ranging from 0.89 to 0.95; mean *H*_*E*_: 0.93; and mean A: 10.80; Table 1). The MLST analysis identified 26 haplotypes, including 22 singletons (HDGI: 0.98) (Table 1 and Fig. 1B). ST6 and ST24 from Reunion and ST17 and ST2 from Cameroon were grouped, respectively, into CC, suggesting genetically close haplotypes for each CC.

At a regional scale (P20), all loci were polymorphic (*H*_*E*_ ranging from 0.36 to 0.74; mean *H*_*E*_: 0.53; and mean A: 4.58). The 20 isolates were resolved into 11 haplotypes with 3 singletons and 2 CCs containing ST18 and ST7 from AEZ4 and AEZ5; ST2, ST3, ST4, ST13, ST14 and ST17 from AEZ3 and AEZ4; (HGDI: 0.91) (Table 1), respectively.

At a local scale (P17), all seven loci were monomorphic.

Altogether, the 65 isolates (C65) were resolved in 32 haplotypes with MLST, including 22 singletons. The discriminatory index HGDI was 0.91. Based on the MLST data (Fig. 1B), the genetic relatedness of the RSSC phylotype III strains generally reflected their geographical origin or structure; some haplotypes from Cameroon (ST28, ST7, ST18 and ST8) and Zimbabwe (ST20 and ST30) were exceptions. Three clonal complexes (MLST-CC1, MLST-CC2, and MLST-CC3) were identified; each complex was composed of strains with a single geographic origin (Cameroon and Reunion, respectively) (Fig. 1B). Grouped haplotypes in MLST-CC1 (ST2, ST3, ST4, ST13, ST14, and ST17) from geographically close Cameroon localities (AEZ3 and AEZ4) were closely related, sharing alleles at 6 of the 7 loci (or SLV); the data are shown in Fig. 1B. ST2 was a common haplotype for strains originating from AEZ3 and AEZ4 in Cameroon; the five remaining haplotypes may originate from this haplotype. MLST-CC2 was an SLV between the Reunion haplotypes ST6 and ST24. MLST-CC3 was composed of ST7 and ST18, which varied at a single locus. Single-field strains from Mfou (AEZ3) and strains from AEZ5 shared the haplotype ST7 (Fig. 1B). This result suggests that the Mfou (AEZ3) and AEZ5 strains were similar and had a common origin. Mfou (AEZ3) strains might have been recently introduced to the surveyed field by clonal expansion or polyclonal introduction. MLST-CC3 was distantly related to ST8 and ST28 from Cameroon and MLST-CC1. The MLST markers are relatively neutral housekeeping genes that evolved slowly instead of under diversifying selective pressures (the locus *egl* is an exception). This may explain the phylogenetic relationships among related Cameroon strains and among Reunion strains. Each MLST-CC is most likely derived from a common ancestor. This complex most likely spread with clonal expansion, and a new variant arose from the source ST and propagated new clonal STs. Typically, haplotypes from Guinea and Zimbabwe were distantly related and differed by four to six loci within their respective countries (ST11 and S15 from Guinea were exceptions that differed at two loci (DLV) (Fig. 1B); however, ST30 from Zimbabwe was 6 LVs from Reunion. Connections by 6 LVs were observed between strains originating from Cameroon (ST13) and Zimbabwe (ST27)/Angola (ST19)/Madagascar (ST22). Haplotypes from Zimbabwe (ST21) and Kenya (ST16) differed by 6 loci, and haplotypes from Ivory Coast (ST5) and Burkina Faso (ST9) also differed by 6 loci. ST5 from the Ivory Coast was 4 LVs from Zimbabwe (ST21) (Fig. 1B). Higher LV numbers suggest that strains are very diverse, distant from each other, and epidemiologically unrelated within a country and between countries. Haplotypes from Madagascar and Reunion Island were closely related genetically, with distances of 2 to 3 LVs. This close genetic relationship was already observed in previous studies involving the partial sequencing of *egl* genes (Poussier et al., 2000a), AFLP analysis (Poussier et al., 2000b), and MLSA analysis (Castillo & Greenberg, 2007; Wicker et al., 2012).

### Complementary input of MLST and RS3-MLVA16 schemes to assess the phylogeny and molecular epidemiology of RSSC phylotype III strains from Africa

The MLVA profiles for the 48 haplotypes generated by RS3-MLVA16 and the MLST profiles of the 32 haplotypes resolved by MLST are listed in Table S1 in the supplemental information. The two typing methods reported a high level of haplotype diversity at the global scale (P35) and the regional scale (P20). However, no diversity was found with MLST at the local scale (P17), whereas RS3-MLVA16 resolved the clonal strains in P17 into 4 haplotypes (Table 1). Although allelic richness and the diversity index generated by MLST were greater, except for P17, compared to those obtained by RS3-MLVA16, resulting in highly polymorphic MLST loci, RS3-MLVA16 had better resolving capacity than MLST according to the high discriminatory ability indices HGDI, number of haplotypes, and singletons observed in all populations surveyed (Table 1). Considering all isolates used in this study (C65), MST analyses (Fig. 1) showed that RS3-MLVA16 could subtype 7 haplotypes unresolved by MLST (ST1, ST2, ST3, ST4, ST6, ST7, and ST17) into MLVA haplotypes (MTs) (Fig. 1A). ST1 was subtyped into MT39, MT41, and MT42 (Guinea); ST2 was subtyped into MT16, MT27, MT31, MT32 and MT43 (Cameroon); ST3 was subtyped into MT15, MT18 and MT19 (Cameroon); ST4 was subtyped into MT16 and MT17 (Cameroon); ST6 was subtyped into MT8, MT9, and MT48 (Reunion); ST7 was subtyped into MT3, MT4, MT5, MT6, and MT47 (Cameroon); and ST17 was subtyped into MT25, MT28, and MT34 (Cameroon). Because TR loci are considered to be rapidly evolving markers, the pattern of locus variation between MTs for each ST clone (which was greater than DLV except for MT4, MT5, MT6, and MT47 on the one hand and MT26 and MT17 on the other hand) reflected their rapid short-term evolution. The SLVs between MT haplotypes in ST7 suggest recent clonal expansion in the Mfou field (AEZ3, Cameroon) and epidemiologically related strains; the source could be MT4. MT3 (ST7) represented in AEZ4 (Cameroon) was 3 LVs from MT4. ST2, which was shared between AEZ3 and AEZ4, and ST6 from Reunion also exhibited recent clonal expansion. MLST-CC1, found in Cameroon, was also differentiated into MTs by RS3-MLVA16 (Fig. 1A); the variation in the MTs was greater than in the DLVs (except MT26 and MT27, which were SLV). Although the Cameroon haplotypes had a close genetic relationship, the strains were clearly unrelated epidemiologically (except for MT16 and MT27). The structure revealed by the MST generated from RS3-MLVA data emphasized high loci variation, which resulted in genetic diversity within the AEZs in Cameroon.

The congruence between the two techniques was different depending on the population analysed. At the global scale (P35), RS3-MLVA16 and MLST were slightly congruent. The aR coefficient was estimated at 0.35 (95% CI, 0.00–1.00), and the genetic distance matrices between the two methods were 0.15 (Mantel’s correlation coefficient, *p*-value = 0.05). Coherence occurs primarily at the level of clonal clusters found in Reunion, Cameroon (AEZ4), and Ivory Coast. However, RS3-MLVA16 showed higher differentiation of the worldwide strains than MLST; RS3-MLVA16 predicted the STs in 74.4% (95% CI, 0.49–1.00), whereas MLST had a lower prediction of partition by RS3-MLVA16 (aW: 22.6%; 95% CI, 0.00–0.46). At the regional scale (P20), the genetic distances between the two methods were highly correlated (Mantel’s correlation coefficient: 0.77, *p*-value < 0.001), but they showed different partitions (aR was 0.00). RS3-MLVA16 was more discriminative and had high prediction of STs (aW: 1.00; 95% CI, 1.00–1.00). At the local scale (P17), RS3-MLVA16 and MLST were not similar, resulting in higher resolution of closely related strains by RS3-MLVA16 (ST7 subtyped into MT4, MT5, MT6 and MT47). Combining the 65 isolates (C65), the congruence between RS3-MLVA16 and MLST was high (Mantel’s correlation coefficient: 0.56, *p*-value < 0.001; aR was 0.56; 95% CI, 0.32–0.84). The MT to predict ST was 0.84 (95%CI, 0.60–1.00), and the ST to predict MTs was 0.50 (95 CI%, 0.18–0.66). Although the correlation between RS3-MLVA16 and MLST was high, differences in the distributions of various haplotypes were observed. MLST structure was not fully maintained in the MST of RS3-MLVA16. Haplotypes from Zimbabwe and Guinea were scattered somewhat by the presence of haplotypes from Cameroon. RS3-MLVA16 confirmed that these haplotypes were epidemiologically unrelated. The Cameroon haplotypes were 5–6 LVs from the Zimbabwean haplotypes. The Guinean haplotypes were 8–10 LVs from strains from other countries (Zimbabwe/Kenya/Cameroon/Ivory Coast; Fig. 1A). The haplotype from Burkina Faso (MT23) was 8 LVs from the Cameroon (MT36) and Ivory Coast (MT1) haplotypes, as determined by RS3-MLVA16. The close genetic relationship between Reunion and Malagasy haplotypes observed by MLST data were not consistent with RS3-MLVA16 (Fig. 1B) and the findings of previous studies (Poussier et al., 2000a; Poussier et al., 2000b; Castillo & Greenberg, 2007; Wicker et al., 2012). This incoherence can result from the difference of evolutionary histories between TR markers and MLST markers. Furthermore, MLST resolved MTs; identical MTs showed different STs: MT2 showed 2 STs (ST11 and ST15), and MT4 combined ST7 and ST18. In such a situation, we cannot exclude the homoplasy effect on some TR loci. A previous study highlighted the inconsistency of trees built from DNA sequences and TR loci due to homoplasy that can arise through convergent/reverse evolution or horizontal gene transfer (Comas et al., 2009). The results underline the high capacity of RS3-MLVA16 for strain identification at global, regional, and local scales. RS3-MLVA16 also underlines genetic variability within the same ST and CC and gives a first overview of population structure.

The ability of a genotyping method to differentiate one RSSC phylotype III strain from another and to delineate their relatedness enables us to unravel the evolutionary pattern and biology of RSSC phylotype III strains from Africa. Assaying genetic variation is critical for understanding the emergence and spread of the RSSC phylotype III across Africa and the Indian Ocean. However, MLST/MLSA has long been considered the gold standard in genotyping for many bacterial pathogens to delineate long-term historical genetic relatedness and to resolve long-standing epidemiological questions, resulting in unambiguous results with nucleotide sequence data (Maiden et al., 1998; Maiden, 2006). However, MLVA typing was successfully applied to investigate the population structure, mostly in short-term and fine-scale epidemiological outbreak investigations and notably in various economically significant monomorphic plant bacterial pathogens. The species analysed include *Xanthomonas citri*, the causative agent of Asiatic citrus canker (Pruvost et al., 2014); *Clavibacter michiganensis*, the causative agent of bacterial wilt and canker in tomato (Zaluga et al., 2013); *Candidatus liberibacter*, the causative agent of the most destructive citrus disease worldwide (Katoh et al., 2011); and *Erwinia amylovora*, the causative agent of a major disease of pome fruit trees (Bühlmann et al., 2014).

In this study, RS3-MLVA16 clearly discerned closely and epidemiologically related strains in field-scale and small-scale analyses, whereas MLST was unable to discriminate sufficiently. RS3-MLVA16, which was based on TR loci, was more capable of recognizing the rapid evolution of strains and was able to describe greater genetic diversity. RS3-MLVA16 provided insight into the regional and local epidemiology of RSSC strains from phylotype III. In this way, RS3-MLVA16 can be used for short-term epidemiology investigations involving local disease outbreaks and routine surveillance. It was observed that the data generated by RS3-MLVA16 complemented the information obtained from MLST. For molecular epidemiology studies, RS3-MLVA16 could be used for the identification of the origin(s) of an inoculum; analysis can also provide insight into the manner in which strains are established in weed reservoirs, the pattern of spread for successful clonal lines, the scale of distribution, and other epidemiological traits associated with the fitness of strains and the extent of disease.

Highly portable, RS3-MLVA16 can be a first-line assay for routine screening at low cost. We are currently using RS3-MLVA16 to investigate these molecular epidemiology traits in bacterial wilt disease caused by RSSC phylotype III strains in Madagascar.

Genotyping data are useful for surveillance networks and outbreak investigations of infectious diseases. Currently, there is not a database for RSSC. The MLST and MLVA databases for RSSC phylotype III recovered from the global, regional, and local populations should be used as a starting point for generating such a database.

## Supplemental Information

10.7717/peerj.1949/supp-1 Figure S1 Muscle alignments of RS2AL03, RS2LB23, RS1L09, and RS3L18 (A) The consensus repeat pattern in the RS2AL03 locus (found on the genome of strain CFBP2957, which belongs to phylotype IIA) is CGGACCTCGCACTT. TRs are not present in the CMR15 genome (a reference strain belonging to phylotype III). (B) The consensus pattern in the RS2LB23 locus (found on the genome of the strain MOLK2, which belongs to phylotype IIB3) is ATCGGTGAC. TRs are absent in the strain CMR15. (C) RS1L09 (found in strain GMI1000, which belongs to phylotype I) matches with RS3L18 (found in the strain CMR15). In strain CMR15, the RS3L18 locus exhibits the following two consecutive TR sequences with different repeat patterns: GTGTCGGCT (9 bp) and GTCAGCGGGATCGGCAGG (18 bp).

10.7717/peerj.1949/supp-2 Figure S2 Visualization of the variability in TR patterns found for RS3L20 in strain CMR15 Mean identity of 51% was observed between repeat units.

10.7717/peerj.1949/supp-3 Figure S3 An illustration of a VNTR loci sequence analysis: alignment of RS3L27 repeats and flanking sequences in four unrelated strains using Muscle RS3L27 is a TR locus with TR units that are 8 bp in length. The lengths of the flanking regions are conserved. The number of TRs differs between different isolates, suggesting that polymorphisms based on size differences are due to variability in the number of repeats. Note the imperfect character of some of the repeats in loci from the CMR15 isolate where an “A” is substituted by a “G”; the single nucleotide differences are observed in the flanking regions and in sequences of the repeated units.

10.7717/peerj.1949/supp-4 Figure S4 HACs of related and unrelated isolates in the three populations of RSSC isolates (P35, P20, P17) and the collection C65genotyped over 16 loci Each circle represents the relationship between the numbers of haplotypes (*g*) detected and the number of loci sampled (*n*). The radius of each circle is equal to the ratio between different combinations of *n* loci detecting *g* haplotypes among the total number of combinations of loci. The bottom and top circles of each bubble-plot indicate the minimum and maximum number of haplotypes found in each population (P35, P20, P17) and in the collection C65. The dotted line represents the upper limit of the maximum number of haplotypes observed.

10.7717/peerj.1949/supp-5 Table S1 Strains of RSSC belonging to phylotype III used in this study and corresponding genotyping data (A) Isolates included in collection P35. (B) Isolates included in collection P20. (C) Isolates included in collection P17. Collection C65 includes all isolates used in this study (*n* = 65). nd: not determined. AEZ: Agro-ecological zones of Cameroon from where RSSC strains originated (N’Guessan et al., 2013).

10.7717/peerj.1949/supp-6 Table S2 Description of TR loci, corresponding oligonucleotide primers and multiplex combinations, and PCR conditions used in this study (A) Nomenclature of VNTR locus was as follows: marker alias_*replicon of origin (ch: chromosome* or *mp: megaplasmid)*_start physical position expressed in kilobases in the CMR15 genome_TR unit size (bp)_ amplicon size in the genome of origin (bp)_number of repeats (units) (e.g., RS1L12_1774_6bp_196bp_12u) (Le Fleche et al., 2002; N’Guessan et al., 2013). Marker alias is RS: R. solanacearum; followed by phylotype number of origin: 1, 2, 3, or 4; and reference of locus (e.g., RS3L12). (B) All positions are in the CMR15 genome. Accession numbers: chromosome ( FP885895) and megaplasmid ( FP885896). (C) Fluorescent dye labelled at 5′-end forward primer.

10.7717/peerj.1949/supp-7 Table S3 Description of MLST loci, corresponding oligonucleotide primers, and PCR conditions (A) Rank according to location within the physical map of the CMR15 genome. Accession numbers: chromosome ( FP885895) and megaplasmid ( FP885896) (B) defined based on the CMR15 genome F: F primer; R: R primer.

10.7717/peerj.1949/supp-8 Table S4 GenBank accession numbers for the partial sequences of MLST loci used in this study Accession numbers of sequences retrieved from GenBank are in italics. Accession numbers of new sequences are in bold.

10.7717/peerj.1949/supp-9 Table S5 Optimal combinations of TR markers used to discriminate all haplotypes from the four collections used (A) The number of possible combinations for each set of loci are given in parentheses. (B) There are 120 possible combinations if 2 of the 16 loci are used, 560 possible combinations if 3 of the 18 loci are used, and so forth. The underlined combinations of loci are the optimal combinations proposed after simulations produced the same maximum number of haplotypes as all 16 loci combined. (C) For example, 4 combinations of 4 loci were optimal for the detection of the 32 haplotypes. Locus RS3L33 discriminated 14 haplotypes among the 32 detected. When combined with locus RS3L17, this 2-locus combination generated 29 haplotypes. Adding locus RS3L27 to the previous combination resolved 31 haplotypes. One of the five proposed loci (RS3L34, RS3L37, or RS3L12) can be added to distinguish all 32 haplotypes. Thus, MLVA based on 4-locus combinations can be used to discriminate all haplotypes in P35. The combination could be RS3L33 + RS3L17 + RS3L27 + RS3L34 (or RS3L37 or RS3L12).

## Additional Information and Declarations

### DNA Deposition

The following information was supplied regarding the deposition of DNA sequences: GenBank: KU255860– KU256216.

### Data Availability

The following information was supplied regarding data availability: Raw data can be found in the Supplemental Information.
