# Supplementary figures and images for: A novel multilocus variable number tandem repeat analysis typing scheme for African phylotype III strains of the Ralstonia solanacearum species complex
Source: PeerJ. 2016 May 5;4:e1949. doi: 10.7717/peerj.1949 (PMC4860299; doi:10.7717/peerj.1949)

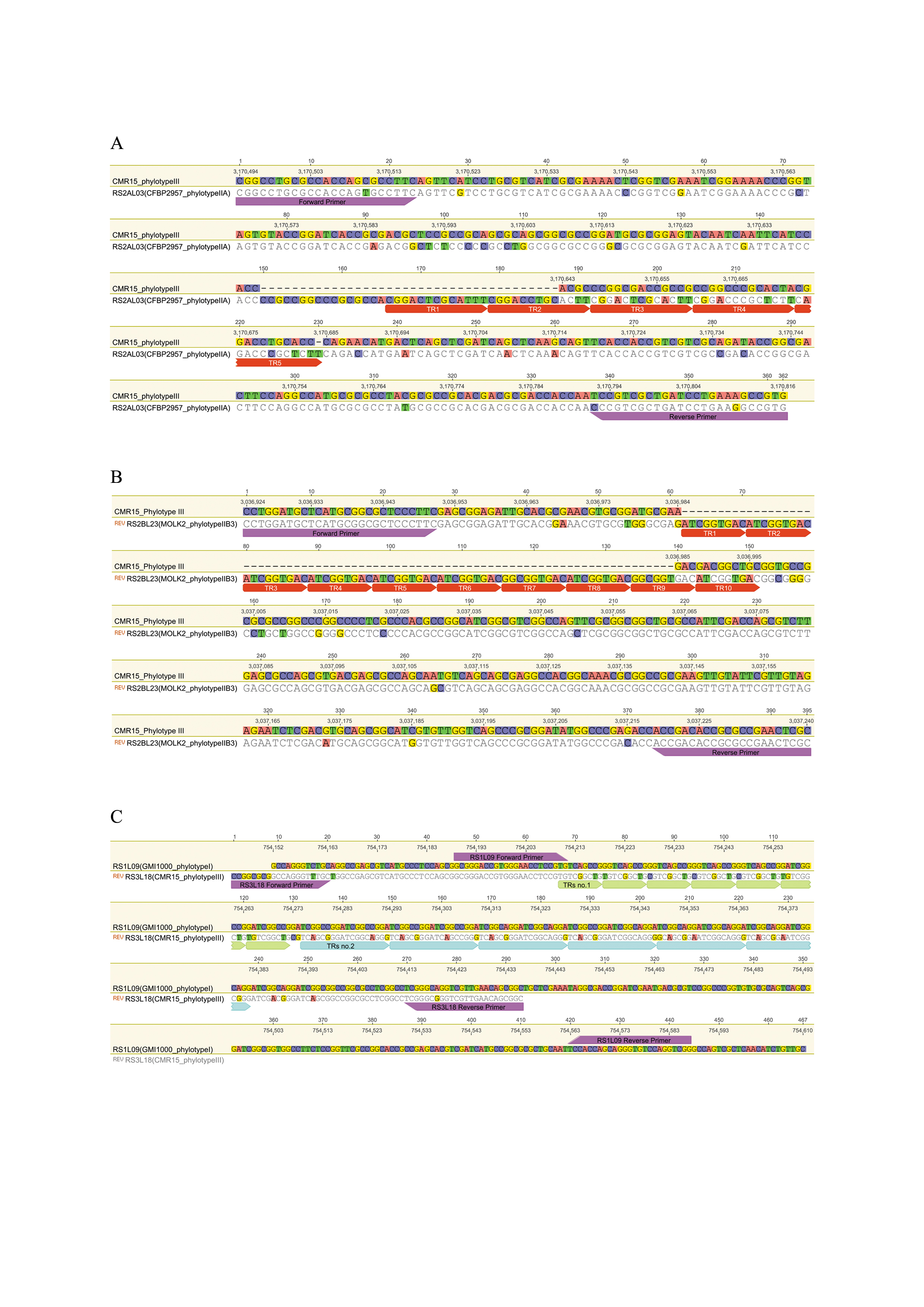

Supplement: Figure S1 — (A) The consensus repeat pattern in the RS2AL03 locus (found on the genome of strain CFBP2957, which belongs to phylotype IIA) is CGGACCTCGCACTT. TRs are not present in the CMR15 genome (a reference strain belonging to phylotype III). (B) The consensus pattern in the RS2LB23 locus (found on the genome of the strain MOLK2, which belongs to phylotype IIB3) is ATCGGTGAC. TRs are absent in the strain CMR15. (C) RS1L09 (found in strain GMI1000, which belongs to phylotype I) matches with RS3L18 (found in the strain CMR15). In strain CMR15, the RS3L18 locus exhibits the following two consecutive TR sequences with different repeat patterns: GTGTCGGCT (9 bp) and GTCAGCGGGATCGGCAGG (18 bp). [file peerj-04-1949-s001.jpg]

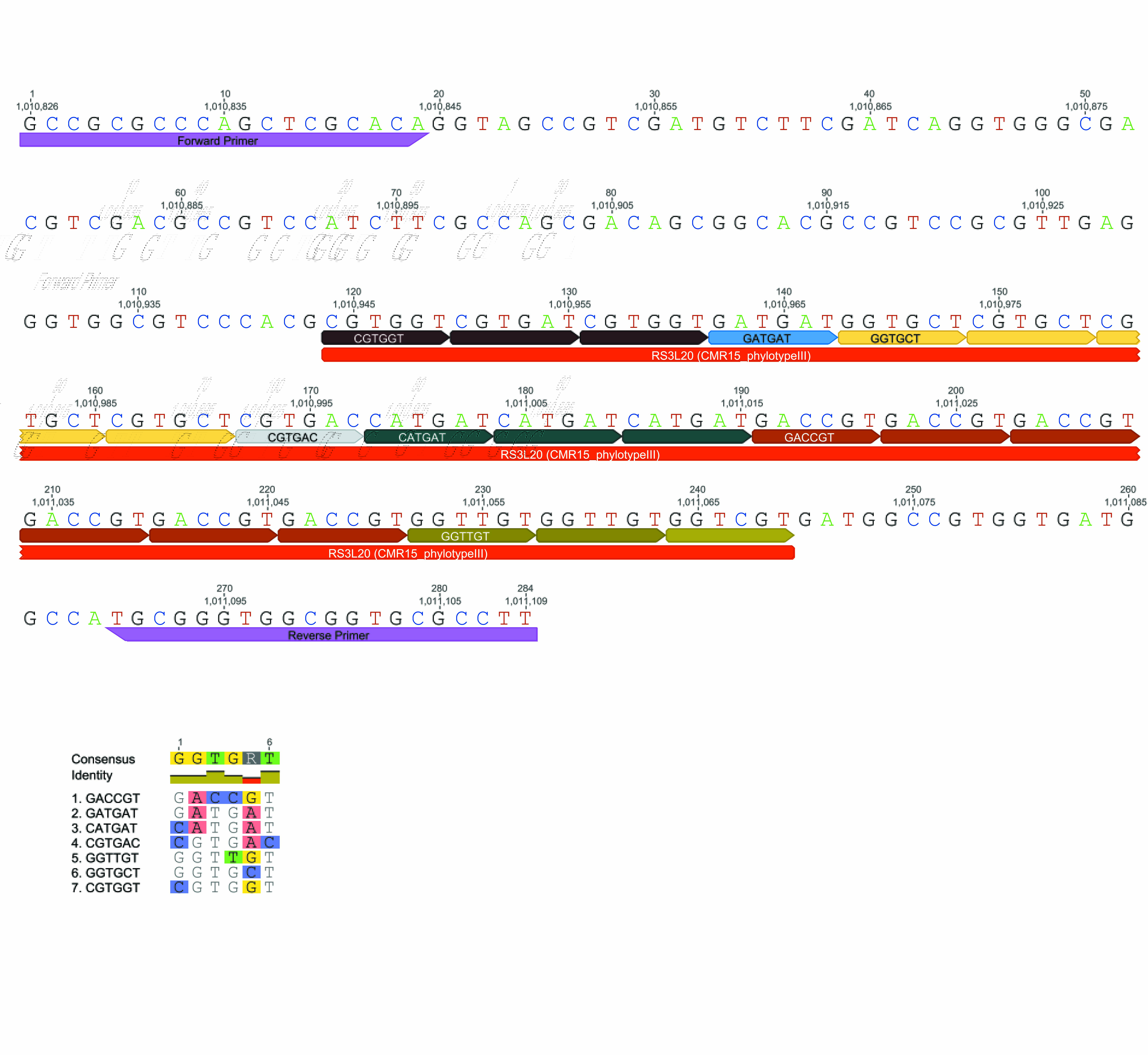

Supplement: Figure S2 — Mean identity of 51% was observed between repeat units. [file peerj-04-1949-s002.jpg]

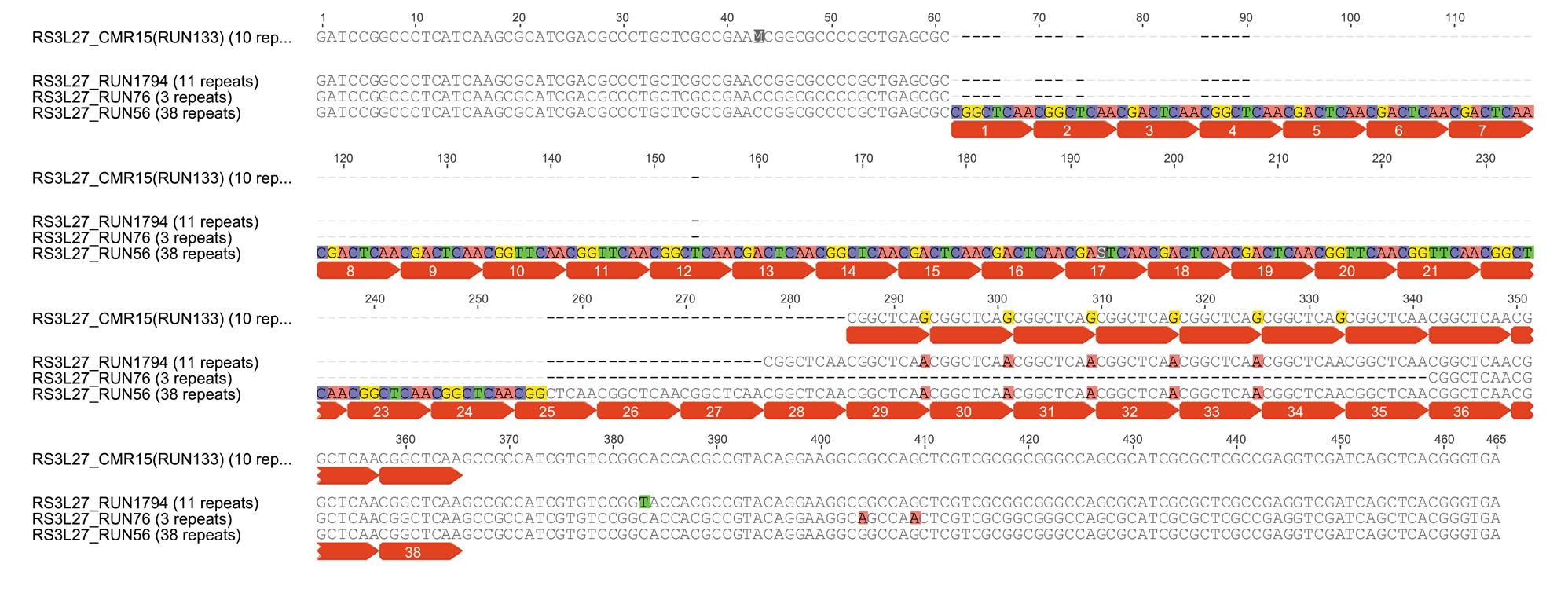

Supplement: Figure S3 — RS3L27 is a TR locus with TR units that are 8 bp in length. The lengths of the flanking regions are conserved. The number of TRs differs between different isolates, suggesting that polymorphisms based on size differences are due to variability in the number of repeats. Note the imperfect character of some of the repeats in loci from the CMR15 isolate where an “A” is substituted by a “G”; the single nucleotide differences are observed in the flanking regions and in sequences of the repeated units. [file peerj-04-1949-s003.jpg]

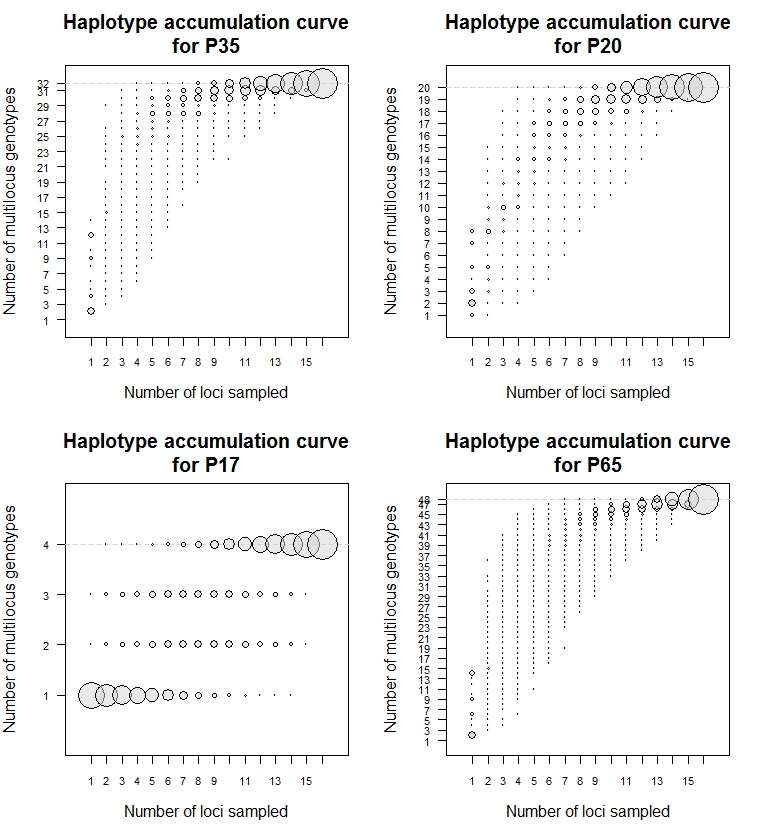

Supplement: Figure S4 — Each circle represents the relationship between the numbers of haplotypes (g) detected and the number of loci sampled (n). The radius of each circle is equal to the ratio between different combinations of n loci detecting g haplotypes among the total number of combinations of loci. The bottom and top circles of each bubble-plot indicate the minimum and maximum number of haplotypes found in each population (P35, P20, P17) and in the collection C65. The dotted line represents the upper limit of the maximum number of haplotypes observed. [file peerj-04-1949-s004.jpeg]
