# Supplementary material for: A novel multilocus variable number tandem repeat analysis typing scheme for African phylotype III strains of the Ralstonia solanacearum species complex
Source: PeerJ. 2016 May 5;4:e1949. doi: 10.7717/peerj.1949 (PMC4860299; doi:10.7717/peerj.1949)
Supplement: Table S4 — Accession numbers of sequences retrieved from GenBank are in italics. Accession numbers of new sequences are in bold. [file peerj-04-1949-s008.pdf]

RUN ID	Strain ID	gyrB	rplB	leuS	adk	mutS	gdhA	egl
RUN0039	CFBP3059	*JF702514*	*JF702124*	*JF702607*	*JF702217*	*AY756766*	*JF702421*	**KU255917**
RUN0046	CIP358	**KU255962**	**KU256166**	**KU256115**	**KU256063**	**KU255860**	**KU256064**	**KU255918**
RUN0053	CFBP7022	**KU255963**	**KU256167**	**KU256116**	**KU256062**	**KU255861**	**KU256065**	*EF439758*
RUN0056	J25	*JF702523*	*JF702133*	*JF702616*	*JF702226*	*AY756810*	*JF702430*	**KU255919**
RUN0060	JT525	*JF702524*	*JF702134*	*JF702617*	*JF702227*	***KU255862***	*JF702431*	**KU255920**
RUN0061	JT528	**KU255964**	**KU256168**	**KU256117**	**KU256061**	**KU255863**	**KU256066**	**KU255921**
RUN0075	NCPPB0332	*JF702530*	*JF702140*	*JF702623*	*JF702233*	**KU255864**	*JF702437*	**KU255922**
RUN0076	NCPPB0342	*JF702531*	*JF702141*	*JF702624*	*JF702234*	*AY756815*	*JF702438*	**KU255923**
RUN0098	K179	*JF702537*	*JF702147*	*JF702630*	*JF702240*	*AY756763*	*JF702444*	**KU255924**
RUN0133	CMR15	*JF702588*	*JF702198*	*JF702681*	*JF702291*	*JF702729*	*JF702495*	**KU255925**
RUN0137	CMR20	**KU255965**	**KU256169**	**KU256118**	**KU256060**	**KU255865**	**KU256067**	*GU295006*
RUN0143	CFBP7028	**KU255966**	**KU256170**	**KU256119**	**KU256059**	**KU255866**	**KU256068**	*EF439748*
RUN0145	CFBP6942	*JF702538*	*JF702148*	*JF702631*	*JF702241*	*EF439773*	*JF702445*	*EF439749*
RUN0146	CMR33	**KU255967**	**KU256171**	**KU256120**	**KU256058**	**KU255867**	**KU256069**	*GU295007*
RUN0149	CFBP7031	**KU255968**	**KU256172**	**KU256121**	**KU256057**	**KU255868**	**KU256070**	*EF439752*
RUN0151	CFBP7033	**KU255969**	**KU256173**	**KU256122**	**KU256056**	**KU255869**	**KU256071**	*EF439753*
RUN0164	CFBP7036	**KU255970**	**KU256174**	**KU256123**	**KU256055**	**KU255870**	**KU256072**	*EF439759*
RUN0165	CFBP7037	**KU255971**	**KU256175**	**KU256124**	**KU256054**	**KU255871**	**KU256073**	*EF439763*
RUN0166	CFBP7038	*JF702546*	*JF702156*	*JF702639*	*JF702249*	*EF439783*	*JF702453*	*EF439729*
RUN0171	CFBP7040	**KU255972**	**KU256176**	**KU256125**	**KU256053**	**KU255872**	**KU256074**	*EF439762*
RUN0172	CFBP7041	**KU255973**	**KU256177**	**KU256126**	**KU256052**	**KU255873**	**KU256075**	*EF439763*
RUN0173	CMR77	**KU255974**	**KU256178**	**KU256127**	**KU256051**	**KU255874**	**KU256076**	**KU255926**
RUN0174	CFBP7042	**KU255975**	**KU256179**	**KU256128**	**KU256050**	**KU255875**	**KU256077**	*EF439764*
RUN0175	CMR79	**KU255976**	**KU256180**	**KU256129**	**KU256049**	**KU255876**	**KU256078**	**KU255927**
RUN0232	CFBP7060	**KU255977**	**KU256181**	**KU256130**	**KU256048**	**KU255877**	**KU256079**	*EF439755*
RUN0233	CFBP7061	**KU255978**	**KU256182**	**KU256131**	**KU256047**	**KU255878**	**KU256080**	*EF439756*
RUN0234	CFBP7062	**KU255979**	**KU256183**	**KU256132**	**KU256046**	**KU255879**	**KU256081**	*EF439757*
RUN0235	CMR53	**KU255980**	**KU256184**	**KU256133**	**KU256045**	**KU255880**	**KU256082**	**KU255928**
RUN0332	MAD029	*JF702558*	*JF702168*	*JF702651*	*JF702261*	**KU255881**	*JF702465*	*GU295041*
RUN0361	DGBBC1139	**KU255981**	**KU256185**	**KU256134**	**KU256044**	**KU255882**	**KU256083**	**KU255929**
RUN0362	DGBBC1138	*JF702562*	*JF702172*	*JF702655*	*JF702265*	**KU255883**	*JF702469*	*GU295009*
RUN0364	DGBBC1227	*JF702563*	*JF702173*	*JF702656*	*JF702266*	**KU255884**	*JF702470*	*GU295011*
RUN0365	DGBBC1136	**KU255982**	**KU256186**	**KU256135**	**KU256043**	**KU255885**	**KU256084**	**KU255930**
RUN0366	DGBBC1222	**KU255983**	**KU256187**	**KU256136**	**KU256042**	**KU255886**	**KU256085**	**KU255931**
RUN0367	DGBBC1223	**KU255984**	**KU256188**	**KU256137**	**KU256041**	**KU255887**	**KU256086**	**KU255932**
RUN0368	DGBBC1259	**KU255985**	**KU256189**	**KU256138**	**KU256040**	**KU255888**	**KU256087**	**KU255933**
RUN0369	DGBBC1125	*JF702564*	*JF702174*	*JF702657*	*JF702267*	**KU255889**	*JF702471*	*GU295008*
RUN0477	CFBP734	**KU255986**	**KU256190**	**KU256139**	**KU256039**	**KU255890**	**KU256088**	**KU255934**
RUN0478	NCPPB0283	**KU255987**	**KU256191**	**KU256140**	**KU256038**	**KU255891**	**KU256089**	**KU255935**
RUN0479	NCPPB1018	*JF702570*	*JF702180*	*JF702663*	*JF702273*	*AY756772*	*JF702477*	**KU255936**
RUN0480	NCPPB0505	**KU255988**	**KU256192**	**KU256141**	**KU256037**	**KU255892**	**KU256090**	**KU255937**
RUN0657	CFBP2146	**KU255989**	**KU256193**	**KU256142**	**KU256036**	**KU255893**	**KU256091**	**KU255938**
RUN0842	CFBP4809	**KU255990**	**KU256194**	**KU256143**	**KU256035**	**KU255894**	**KU256092**	**KU255939**
RUN1238	CFBP4963	**KU255991**	**KU256195**	**KU256144**	**KU256034**	**KU255895**	**KU256093**	**KU255940**
RUN1298	JS949	**KU255992**	**KU256196**	**KU256145**	**KU256033**	**KU255896**	**KU256094**	**KU255941**
RUN1793	CIV56	**KU255993**	**KU256197**	**KU256146**	**KU256032**	**KU255897**	**KU256095**	**KU255942**
RUN1794	CIV57	**KU255994**	**KU256198**	**KU256147**	**KU256031**	**KU255898**	**KU256096**	**KU255943**
RUN1796	CIV59	**KU255995**	**KU256199**	**KU256148**	**KU256030**	**KU255899**	**KU256097**	**KU255944**
RUN3592	TM-T1-CM01A	**KU255996**	**KU256200**	**KU256149**	**KU256029**	**KU255900**	**KU256098**	**KU255945**
RUN3593	TM-T1-CM01B	**KU255997**	**KU256201**	**KU256150**	**KU256028**	**KU255901**	**KU256099**	**KU255946**
RUN3594	TM-T1-CM02A	**KU255998**	**KU256202**	**KU256151**	**KU256027**	**KU255902**	**KU256100**	**KU255947**
RUN3595	TM-T1-CM02B	**KU255999**	**KU256203**	**KU256152**	**KU256026**	**KU255903**	**KU256101**	**KU255948**
RUN3596	TM-T1-CM06A	**KU256000**	**KU256204**	**KU256153**	**KU256025**	**KU255904**	**KU256102**	**KU255949**
RUN3597	TM-T1-CM06B	**KU256001**	**KU256205**	**KU256154**	**KU256024**	**KU255905**	**KU256103**	**KU255950**
RUN3598	TM-T1-CM11A	**KU256002**	**KU256206**	**KU256155**	**KU256023**	**KU255906**	**KU256104**	**KU255951**
RUN3599	TM-T1-CM11B	**KU256003**	**KU256207**	**KU256156**	**KU256022**	**KU255907**	**KU256105**	**KU255952**
RUN3600	TM-T1-CM20	**KU256004**	**KU256208**	**KU256157**	**KU256021**	**KU255908**	**KU256106**	**KU255953**
RUN3601	TM-T1-CM21	**KU256005**	**KU256209**	**KU256158**	**KU256020**	**KU255909**	**KU256107**	**KU255954**
RUN3602	TM-T2-CM06	**KU256006**	**KU256210**	**KU256159**	**KU256019**	**KU255910**	**KU256108**	**KU255955**
RUN3603	TM-T2-CM07A	**KU256007**	**KU256211**	**KU256160**	**KU256018**	**KU255911**	**KU256109**	**KU255956**
RUN3604	TM-T2-CM07B	**KU256008**	**KU256212**	**KU256161**	**KU256017**	**KU255912**	**KU256110**	**KU255957**
RUN3605	TM-T2-CM16	**KU256009**	**KU256213**	**KU256162**	**KU256016**	**KU255913**	**KU256111**	**KU255958**
RUN3606	TM-T2-CM17	**KU256010**	**KU256214**	**KU256163**	**KU256015**	**KU255914**	**KU256112**	**KU255959**
RUN3607	TM-T2-CM18A	**KU256011**	**KU256215**	**KU256164**	**KU256014**	**KU255915**	**KU256113**	**KU255960**
RUN3608	TM-T2-CM18B	**KU256012**	**KU256216**	**KU256165**	**KU256013**	**KU255916**	**KU256114**	**KU255961**
