# Supplementary material for: A novel multilocus variable number tandem repeat analysis typing scheme for African phylotype III strains of the Ralstonia solanacearum species complex
Source: PeerJ. 2016 May 5;4:e1949. doi: 10.7717/peerj.1949 (PMC4860299; doi:10.7717/peerj.1949)
Supplement: Table S5 — (A) The number of possible combinations for each set of loci are given in parentheses. (B) There are 120 possible combinations if 2 of the 16 loci are used, 560 possible combinations if 3 of the 18 loci are used, and so forth. The underlined combinations of loci are the optimal combinations proposed after simulations produced the same maximum number of haplotypes as all 16 loci combined. (C) For example, 4 combinations of 4 loci were optimal for the detection of the 32 haplotypes. Locus RS3L33 discriminated 14 haplotypes among the 32 detected. When combined with locus RS3L17, this 2-locus combination generated 29 haplotypes. Adding locus RS3L27 to the previous combination resolved 31 haplotypes. One of the five proposed loci (RS3L34, RS3L37, or RS3L12) can be added to distinguish all 32 haplotypes. Thus, MLVA based on 4-locus combinations can be used to discriminate all haplotypes in P35. The combination could be RS3L33 + RS3L17 + RS3L27 + RS3L34 (or RS3L37 or RS3L12). [file peerj-04-1949-s009.pdf]

no. loci	1	2	3	4	5	6	7
no. possible combination [a]	*(16)*	*(120)* [b]	*(560)*	*(1820)*	*(4368)*	*(8008)*	*(11440)*
**Population P35** [c]							
Combination of loci	RS3L33<br>or RS3L33<br>or RS3L33	+ RS3L17<br>+ RS3L17<br>+ RS3L17	+ RS3L27<br>+ RS3L27<br>+ RS3L27	+ RS3L34<br>+ RS3L37<br>+ RS3L12	-	-	-
Haplotypes detected	*14*	*29*	*31*	32			
**Population P20**							
Combination of loci	RS1L12<br>or RS3L37	+ RS3L33<br>+ RS3L33	+ RS3L17<br>+ RS3L17	+ RS3L36<br>+ RS3L36	-	-	-
Haplotypes detected	*8*	*15*	*18*	*20*			
**Population P17**							
Combination of loci	RS3L30	+ RS3L28	-	-	-	-	-
Haplotypes detected	*2*	*4*					
**Collection C65**							
Combination of loci	RS3L17<br>or RS3L37	+ RS3L33<br>+ RS3L33	+ RS3L30<br>+ RS3L30	+ RS1L12<br>+ RS1L12	+ RS3L27<br>+ RS3L27	+ RS3L28<br>+ RS3L28	+ RS3L36<br>+ RS3L36
Haplotypes detected	*14*	*36*	*41*	*45*	*46*	*47*	*48*
